# Plasma proteomics based on machine learning predict the early risk of kidney outcomes in patients with DKD: a prospective cohort study from UK Biobank

**DOI:** 10.3389/fneph.2026.1862772

**Published:** 2026-06-29

**Authors:** Li Jiang, Tingting Li, Haojun Zhang, Xiai Wu, Tingting Zhao

**Affiliations:** 1 Diabetes Department of Integrated Chinese and Western Medicine, China National Center for Integrated Traditional Chinese and Western Medicine, China-Japan Friendship Hospital, Beijing, China; 2 Department of Colorectal Surgery, China National Center for Integrated Traditional Chinese and Western Medicine, China-Japan Friendship Hospital, Beijing, China; 3 Institute of Clinical Medical Sciences, China-Japan Friendship Hospital, Beijing, China

**Keywords:** diabetic kidney disease, early risk stratification, kidney outcomes, machine learning, plasma proteomics, predictive biomarkers

## Abstract

**Background:**

Diabetic kidney disease (DKD) is the predominant cause of end-stage kidney disease worldwide. Early identification of patients at heightened risk for adverse kidney outcomes remains a critical unmet clinical need.

**Methods:**

We conducted a prospective cohort study of 918 DKD participants from the UK Biobank. Baseline plasma proteomic profiling quantified 1,463 proteins using Olink proximity extension assays. Three nested Cox proportional hazards models with incremental covariate adjustment identified proteins associated with composite kidney outcomes. To prevent information leakage, all feature selection, hyperparameter optimization, and model development were performed exclusively within the training cohort (70% random split) via a multi-stage pipeline integrating LASSO-Cox regression, Random Survival Forest, Boruta algorithm, and Sequential Forward Selection. XGBoost Cox modeling with SHAP interpretability analysis quantified variable contributions. Predictive performance was validated through Kaplan–Meier survival analysis, 15-year longitudinal trajectory modeling, ROC benchmarking, 10-fold nested cross-validation, and sensitivity analyses restricted to KDIGO-defined DKD. An interactive web application was developed for clinical translation.

**Results:**

Of 1,463 proteins examined, 633 demonstrated significant associations with kidney outcomes across all three Cox models. Functional enrichment highlighted immune-inflammatory pathways, PI3K-Akt signaling, and chemokine cascades as central to DKD progression. The machine learning framework identified an 11-protein signature, which was refined to 10 core biomarkers (HLA-E, EFNA1, GPR158, FSTL3, ART3, GM2A, CLEC1A, CKAP4, IFNGR1, EPHA2) following survival and longitudinal trajectory validation. The protein-only model achieved robust discrimination (AUC = 0.808 [95% CI 0.767–0.848] for composite outcomes; AUC = 0.807 [95% CI 0.765–0.848] for renal death), comparable to fully integrated models incorporating demographics and metabolic variables. Systematic benchmarking across 101 algorithm combinations identified LASSO-RSF as optimal (C-index = 0.94 in training; 0.73 in independent testing), with reliable calibration through mid-term follow-up (Integrated Calibration Index = 0.019–0.022). The web-based tool enables real-time, personalized risk stratification.

**Conclusions:**

This study establishes a validated 10-protein signature for early prediction of kidney outcomes in DKD. The systematic machine learning framework and deployable web application provide accessible, interpretable risk assessment to support precision nephrology and preemptive clinical intervention.

## Introduction

Diabetic kidney disease (DKD) is one of the most formidable complications of diabetes mellitus and the leading cause of end-stage kidney disease requiring renal replacement therapy worldwide (1). The escalating prevalence of type 2 diabetes has precipitated a parallel surge in DKD incidence, imposing heavy burdens on healthcare systems and profoundly compromising quality of life for affected individuals (2). Despite advances in glycemic control and renin-angiotensin-aldosterone system inhibition, many DKD patients experience inexorable disease progression, underscoring the imperative for novel prognostic tools capable of identifying high-risk individuals during the window when interventions might prove most efficacious (3).

Contemporary clinical practice relies predominantly on albuminuria and estimated glomerular filtration rate as sentinel markers of renal impairment (4). While these parameters provide valuable information regarding current kidney function, they exhibit limitations in predicting future disease trajectory. Albuminuria demonstrates considerable intraindividual variability, and eGFR decline often reflects established structural damage rather than impending risk (5). Emerging biomarkers including kidney injury molecule-1 and neutrophil gelatinase-associated lipocalin have shown promise in detecting acute kidney injury, yet their utility for long-term prognostication in chronic DKD remains less well established (6, 7). Several validated clinical risk prediction tools have been developed for chronic kidney disease, such as the Kidney Failure Risk Equation (KFRE), which estimates the 2- and 5-year probability of progression to kidney failure based on age, sex, eGFR, and albuminuria (8). However, its predictive performance may be attenuated in specific clinical contexts, including early-stage DKD, where the equation exhibits greater sensitivity to known within-person variability in urinary albumin-to-creatinine ratio (UACR) and eGFR inputs (9).

Plasma proteomics offers a transformative approach to biomarker discovery, enabling simultaneous interrogation of hundreds of circulating proteins that reflect diverse pathophysiological processes including inflammation, metabolism, endothelial dysfunction, and tissue remodeling (10). Onoja et al. identified a three-protein panel (sTNFR1, sCD40, COL1A1) with discrimination comparable to established risk factors for kidney failure (11), while Dubin et al. analyzed associations of 4,638 plasma proteins among 3,235 participants of the Chronic Renal Insufficiency Cohort Study, identifying modifiable markers associated with CKD progression (12), and Ramírez Medina et al. implicated complement cascade activation as a proteomic signature of diabetic nephropathy progression (13). The high-dimensional nature of proteomic data, however, exceeds the analytical capacity of conventional statistical methods, necessitating integration with advanced machine learning algorithms capable of capturing complex, non-linear relationships and interactions among variables (14). Ensemble methods including LASSO regression, Random Survival Forest, and gradient boosting have demonstrated particular utility in distilling robust predictive signatures from high-dimensional omics datasets (15).

In this prospective cohort investigation, we leveraged large-scale plasma proteomics from the UK Biobank to identify protein biomarkers predictive of kidney outcomes in DKD patients. Through a systematic multi-stage machine learning framework, we distilled an initial panel of 633 candidate proteins to a validated 10-protein signature. We further developed an interactive web-based application to facilitate clinical translation of these findings.

## Methods

### Study population

The UK Biobank comprises a prospective, population-based cohort of approximately 500,000 adults aged 39–70 years recruited from 22 assessment centers throughout the United Kingdom between 2006 and 2010 (16). This comprehensive resource prospectively links participant records to nationwide mortality, hospitalization, and cancer registries. For a probability subsample, high-throughput plasma proteomics were measured using the Olink Explore 1536 panel assay.

We identified DKD cases through an integrative approach combining diagnostic codes from prior studies (17) and established clinical guidelines (18). Eligibility criteria encompassed: (1) medical record documentation of ICD-10 codes indicating diabetic renal complications (E10.2, E11.2, E14.2, N08.3); (2) ICD-9 assignment 250.4 for diabetes mellitus with renal manifestations; or (3) for patients with confirmed diabetes, fulfillment of any subsidiary condition including CKD-specific ICD-10 coding (N18.0-N18.9) or OPCS-4 procedural codes (M01, X40), baseline self-report of chronic kidney disease or renal failure, or laboratory values demonstrating eGFR below 60 mL/min/1.73m² and/or urinary albumin-to-creatinine ratio ≥30 mg/g sustained for at least three months(checking between instance 0, 1 and 2, if there were no records of repeated measurements, then refer to the eGFR based on a single measurement). Diabetes ascertainment employed multiple criteria: physician diagnosis, self-reported condition, current antidiabetic medication use, HbA1c exceeding 48 mmol/mol, or fasting glucose above 7.0 mmol/L. Detailed diagnostic codes are provided in **Supplementary Table 1**.

From an initial cohort of 3,213 individuals with DKD, we sequentially excluded participants with pre-existing kidney failure or ongoing replacement therapy at baseline, those lacking proteomic data, and individuals failing quality control criteria. The final analytical sample comprised 918 DKD patients. During follow-up, 779 patients remained event-free while 139 experienced incident composite kidney outcomes, including 127 primary outcomes (renal death) and 16 secondary outcomes (**Supplementary Figure 1**).

### Kidney outcome definitions

The composite kidney outcome encompassed four distinct components: (1) renal death (primary outcome), defined as mortality directly attributable to renal failure or associated kidney diseases; Ascertained through linked national death registry records (UK Biobank fields 40001 and 40002) using ICD-10 codes for underlying cause of death, specifically N00–N19. (2) kidney failure (end-stage kidney disease), characterized by initiation of chronic dialysis, renal transplantation, or sustained eGFR below 15 mL/min/1.73m²; (3) substantial eGFR decline, defined as ≥40% decrease from baseline sustained for at least 28 days; and (4) albuminuria progression, operationalized as ≥30% increase in UACR from baseline sustained for at least two years (19, 20). The decline in eGFR or the progression of urinary protein were identified through the repeated assessment of consecutive serum creatinine measurements and UACR measurements during the visits (instance 0, 1 and 2).The data extraction was conducted on December 2025 (version v20). Events were captured through linkage to national mortality registries, primary care documentation, hospital admission statistics, and participant self-reports. Follow-up extended from enrollment to the first occurrence of any outcome component, death, or censoring at study termination.

### Plasma proteomics

Baseline blood specimens were collected at 22 assessment centers between 2007 and 2010. Following EDTA anticoagulation, samples were centrifuged at 2500 g for 10 minutes at 4 °C, with plasma aliquots stored at -80 °C and shipped on dry ice to the Olink core facility in Sweden. High-sensitivity proximity extension immunoassays quantified 1,463 cardiovascular, metabolic, inflammatory, neurological, and oncology-related proteins expressed as log2-transformed normalized protein expression values (21). Intra- and inter-plate coefficients of variation were maintained below 10% and 20%, respectively. Laboratory personnel remained blinded to all clinical data throughout processing.

### Clinical variables

We extracted comprehensive baseline characteristics encompassing demographic fundamentals (age, sex, ethnicity, Townsend deprivation index, education score), anthropometric measurements (BMI, waist circumference), glycemic parameters (HbA1c, fasting glucose), renal function markers (eGFR, UACR, serum urea, cystatin C), lipid profiles (HDL, LDL, triglycerides, apolipoprotein B, lipoprotein A), blood pressure readings, inflammatory markers (C-reactive protein), and lifestyle factors (smoking status, alcohol consumption). Detailed variable definitions appear in **Supplementary Table 2**.

### Cox regression analysis

Cox proportional hazards models with follow-up duration as the temporal axis estimated hazard ratios and 95% confidence intervals for associations between plasma proteins and kidney outcomes (22). Three nested models with incremental covariate adjustment were constructed: Model 1 (crude) contained no covariates; Model 2 (demographic) controlled for age, sex, ethnicity, education score, and Townsend deprivation index; Model 3 (fully adjusted) further incorporated HbA1c, glucose, lipid parameters, urea, BMI, eGFR, UACR, blood pressure, and waist circumference. To mitigate multicollinearity, variables with variance inflation factor exceeding 10 were excluded (**Supplementary Figure 2**). Benjamini-Hochberg false discovery rate correction was applied across all protein-outcome associations. Statistical significance was established at two-sided adjusted P < 0.05. Proteins significant across all three models were selected for downstream machine learning analysis. This candidate pool was derived from the entire cohort exclusively for exploratory enrichment and descriptive purposes; no feature selection or model construction was performed at this stage.

### Functional enrichment analysis

Candidate proteins demonstrating significant associations were aggregated and converted to Entrez gene identifiers for pathway interrogation using clusterProfiler and org.Hs.eg.db packages (23). Gene Ontology annotation examined biological processes, cellular components, and molecular functions. KEGG pathway mapping identified enriched signaling cascades (24). Significance thresholds were set at nominal P < 0.05 with false discovery rate below 0.20.

### Machine learning framework

To prevent information leakage and ensure unbiased model evaluation, the cohort was first randomly partitioned into training (70%) and testing (30%) sets. The training set was used for all feature selection, hyperparameter optimization, and model development described below. The testing set remained entirely unseen until final performance assessment. We constructed a systematic four-stage analytical architecture for protein prioritization applied strictly within the training data: Stage 1: LASSO-Cox Regularization. Penalized Cox regression with L1 regularization shrank less informative variable coefficients toward zero, yielding a parsimonious predictor set while controlling multicollinearity (25). The penalty parameter λ was optimized via 10-fold cross-validation at the one-standard-error criterion. Stage 2: Random Survival Forest. An ensemble of 1,000 decision trees with maximum depth of 8 nodes and terminal node size ≥5 events captured non-linear hazard relationships and generated permutation-derived importance rankings (26). Stage 3: Boruta Algorithm. This all-relevant feature selection method compared real feature importance against permuted shadow features across 100 iterations, retaining only proteins consistently surpassing shadow baselines (27). Stage 4: Sequential Forward Selection. Variables were iteratively added to the model, tracking cumulative C-index changes until predictive performance peaked and subsequently declined, identifying the optimal subset where accuracy was maximized. XGBoost Cox and SHAP Analysis. Gradient boosting with learning rate 0.01, tree depth 3, and 500 rounds refined final selection while SHAP decomposition quantified each protein’s marginal contribution to individualized risk predictions (28).

### Survival and trajectory analyses

Algorithmically prioritized proteins underwent binary partitioning via ROC-optimized cutpoint selection. Kaplan-Meier curves compared event-free survival between expression-level cohorts, with hazard ratios estimated via Cox regression. To account for death as a competing risk, Fine and Gray sub-distribution hazard models estimated cumulative incidence of kidney outcomes (29). Longitudinal trajectories of plasma proteins were modeled over a 15-year period prior to the index date using a landmark analysis framework matched by follow-up time. Smoothing curves were used to visualize temporal trends; Mann--Kendall tests assessed monotonicity. Mixed-effects models were applied to test time-group interactions (30).

### Predictive performance evaluation

Receiver operating characteristic analyses evaluated predictive accuracy of the protein signature alone and in combination with demographic indicators (age, sex, ethnicity, education, Townsend index) and metabolic traits (HbA1c, glucose, lipids, BMI, blood pressure, renal markers). Four sequential models were compared: protein-only, protein plus demographics, protein plus demographics and metabolic factors, and fully integrated models. DeLong tests and bootstrap analyses with 2,000 iterations assessed AUC differences (31). To mitigate overfitting, the cohort was randomly partitioned into training (70%) and testing (30%) sets, with repeated cross-validation performed. To further assess the robustness of the protein signature, a sensitivity analysis was performed by restricting the cohort to participants who fulfilled the KDIGO laboratory-based definition of chronic kidney disease (persistent eGFR <60 mL/min/1.73 m² or urinary albumin-to-creatinine ratio ≥30 mg/g sustained for at least 3 months), with ROC curves and AUC distributions re-evaluated in this subgroup.

Systematic algorithm benchmarking screened 101 machine learning combinations using the Mime framework to identify the optimal modeling approach. The LASSO-RSF framework demonstrating highest C-index performance across both training and independent testing cohorts was selected as the final model.

### Web application development

The validated predictive model was encapsulated as an interactive web application using the Shiny framework. The interface comprises three functional modules: model information display, protein value input with clinical reference ranges, and biomarker annotation. Real-time risk probability calculation enables personalized prognostic assessment.

### Ethical considerations

This investigation was conducted in accordance with the Declaration of Helsinki. All participants provided electronic informed consent before enrollment, and the protocol received approval from the North West Multi-Centre Research Ethics Committee (reference 11/NW/0382). The UK Biobank granted data access under application number 564216.

## Results

### Baseline characteristics

**Table 1** presents baseline characteristics of the 918 DKD participants. The case group (N = 139) demonstrated significantly lower eGFR (53.8 vs 77.1 mL/min/1.73 m², P<0.001), higher UACR (36.2 vs 15.6 mg/g, P = 0.004), elevated urea (10.6 vs 6.82 mmol/L, P<0.001), and reduced albumin (43.0 vs 44.5 g/L, P<0.001) compared to controls (N = 779). Cases also exhibited longer diabetes duration (17.1 vs 10.9 years, P<0.001), higher prevalence of diabetic neuropathy, and more extensive medication use for diabetes and hypertension. Notably, conventional cardiovascular risk factors including BMI, blood pressure, and lipid profiles showed no significant differences between groups, underscoring the need for more sensitive prognostic biomarkers.

**Table 1:** Baseline characteristics of UK Biobank participants included in the study.

	Control	Kidney outcomes	P value (Kidney outcomes vs. Control)	Primary outcomes (renal death)	P value (Primary outcomes vs. Control)	Secondary outcomes	P value (Secondary outcomes vs. Control)
	N=779	N=139		N=127		N=16	
Age (years)	60.7 (7.04)	63.5 (5.36)	<0.001	64.0 (5.02)	<0.001	59.3 (5.79)	0.236
Sex:			0.353		0.352		0.975
Female	276 (35.4%)	43 (30.9%)		39 (30.7%)		5 (31.2%)	
Male	503 (64.6%)	96 (69.1%)		88 (69.3%)		11 (68.8%)	
Ethnicity:			0.558		0.462		0.154
Asian	41 (5.26%)	7 (5.04%)		7 (5.51%)		0 (0.00%)	
Black	34 (4.36%)	5 (3.60%)		3 (2.36%)		3 (18.8%)	
Mixed	1 (0.13%)	0 (0.00%)		0 (0.00%)		0 (0.00%)	
Prefer not to answer	2 (0.26%)	1 (0.72%)		1 (0.79%)		0 (0.00%)	
White	676 (86.8%)	125 (89.9%)		115 (90.6%)		13 (81.2%)	
Townsend deprivation index	-0.02 (3.56)	-0.20 (3.55)	0.593	-0.24 (3.55)	0.516	0.23 (3.84)	0.779
Education score	23.1 (19.5)	22.5 (20.2)	0.789	22.7 (20.3)	0.891	23.0 (20.8)	0.993
eGFR(mL/min/1.73 m²)	77.1 (23.2)	53.8 (21.3)	<0.001	54.1 (20.4)	<0.001	47.7 (27.3)	0.002
ACR(mg/g)	15.6 (36.1)	36.2 (74.9)	0.004	36.8 (77.8)	0.007	35.2 (42.8)	0.139
HbA1c(mmol/mol)	55.5 (18.3)	57.8 (19.3)	0.211	58.4 (19.7)	0.122	53.0 (17.6)	0.558
Diabetes duration(years)	10.9 (11.9)	17.1 (14.8)	<0.001	17.0 (15.2)	<0.001	15.3 (8.23)	0.182
Diabetic neuropathy:			0.006		<0.001		0.442
Do not know	10 (1.28%)	1 (0.72%)		1 (0.79%)		0 (0.00%)	
No	116 (14.9%)	7 (5.04%)		4 (3.15%)		3 (18.8%)	
Yes	25 (3.21%)	4 (2.88%)		3 (2.36%)		1 (6.25%)	
Diastolic blood pressure(mmHg)	83.1 (11.9)	79.0 (12.3)	0.001	78.9 (12.5)	0.001	81.8 (10.3)	0.795
Systolic blood pressure(mmHg)	147 (20.6)	149 (19.4)	0.419	149 (19.6)	0.299	149 (20.0)	0.806
BMI(kg/m²)	32.5 (6.18)	33.3 (5.93)	0.179	33.5 (6.00)	0.096	30.9 (4.46)	0.128
Waist circumference(cm)	106 (14.9)	109 (15.2)	0.030	110 (15.1)	0.010	102 (14.0)	0.218
Hip circumference(cm)	110 (12.6)	111 (12.5)	0.422	112 (12.8)	0.280	108 (7.36)	0.197
HDL cholesterol(mmol/L)	1.13 (0.32)	1.08 (0.31)	0.062	1.05(0.28)	0.133	1.12(0.34)	0.080
Triglycerides(mmol/L)	2.39 (1.42)	2.38 (1.47)	0.931	2.38 (1.50)	0.906	2.54 (1.49)	0.689
LDL cholesterol(mmol/L)	2.75 (0.85)	2.46 (0.82)	<0.001	2.45 (0.84)	<0.001	2.42 (0.60)	0.070
Apolipoprotein B cholesterol(mmol/L)	0.87 (0.24)	0.80 (0.22)	0.001	0.80 (0.23)	0.002	0.75 (0.16)	0.013
Lipoprotein A(mmol/L)	47.6 (51.1)	45.0 (46.8)	0.615	46.4 (47.9)	0.870	28.4 (30.2)	0.064
Glucose(mmol/L)	7.90 (3.91)	8.51 (4.35)	0.148	8.64 (4.42)	0.088	8.16 (3.76)	0.880
Hypertension:			0.179		0.495		0.057
No/Unknown	308 (39.5%)	46 (33.1%)		45 (35.4%)		2 (12.5%)	
Yes	471 (60.5%)	93 (66.9%)		82 (64.6%)		14 (87.5%)	
Hypertension duration(years)	10.7 (8.27)	13.8 (9.02)	0.002	14.2 (9.40)	0.002	10.5 (4.94)	0.614
Urea(mmol/L)	6.82 (2.64)	10.6 (5.91)	<0.001	10.5 (5.61)	<0.001	13.2 (8.45)	0.013
Albumin(g/L)	44.5 (3.17)	43.0 (3.19)	<0.001	43.0 (3.18)	<0.001	42.6 (3.41)	0.091
Cystatin C(mg/L)	1.15 (0.34)	1.67 (0.60)	<0.001	1.66 (0.58)	<0.001	1.78 (0.79)	0.012
Medications for cholesterol:			0.173		0.218		1.000
No	394 (50.6%)	61 (43.9%)		56 (44.1%)		8 (50.0%)	
Yes	385 (49.4%)	78 (56.1%)		71 (55.9%)		8 (50.0%)	
Medications for diabetes:			0.001		0.004		0.305
No	668 (85.8%)	103 (74.1%)		95 (74.8%)		12 (75.0%)	
Yes	111 (14.2%)	36 (25.9%)		32 (25.2%)		4 (25.0%)	
Medications for blood pressure:			<0.001		<0.001		0.423
No	415 (53.3%)	49 (35.3%)		45 (35.4%)		6 (37.5%)	
Yes	364 (46.7%)	90 (64.7%)		82 (64.6%)		10 (62.5%)	
C reactive protein(mg/L)	4.30 (5.93)	5.72 (7.89)	0.050	5.92 (8.17)	0.037	3.49 (3.39)	0.248
Alanine aminotransferase(U/L)	30.7 (21.6)	22.4 (11.8)	<0.001	22.4 (12.1)	<0.001	21.0 (9.49)	0.003
Calcium(mmol/L)	2.39 (0.12)	2.35 (0.09)	<0.001	2.35 (0.09)	<0.001	2.35 (0.13)	0.294
Potassium in urine(mmol/L)	60.6 (30.3)	52.3 (25.8)	0.001	52.7 (26.2)	0.004	49.0 (18.5)	0.040
Sodium in urine(mmol/L)	80.2 (44.6)	67.8 (37.6)	0.001	65.8 (36.2)	<0.001	80.8 (46.3)	0.831
Vitamin D(mmol/L)	39.5 (20.4)	39.9 (19.8)	0.826	40.0 (20.0)	0.805	38.2 (17.2)	0.760
Alcohol drinking status:			0.383		0.391		0.145
Current	638 (81.9%)	108 (77.7%)		99 (78.0%)		11 (68.8%)	
Never	73 (9.37%)	15 (10.8%)		14 (11.0%)		1 (6.25%)	
Prefer not to answer	1 (0.13%)	1 (0.72%)		1 (0.79%)		0 (0.00%)	
Previous	66 (8.47%)	15 (10.8%)		13 (10.2%)		4 (25.0%)	
Smoking status:			0.650		0.659		0.542
Current	84 (10.8%)	12 (8.63%)		12 (9.45%)		0 (0.00%)	
Never	317 (40.7%)	57 (41.0%)		51 (40.2%)		8 (50.0%)	
Prefer not to answer	5 (0.64%)	2 (1.44%)		2 (1.57%)		0 (0.00%)	
Previous	372 (47.8%)	68 (48.9%)		62 (48.8%)		8 (50.0%)	
Sleep hours	7.25 (1.56)	7.74 (1.71)	0.002	7.79 (1.71)	0.001	7.06 (1.57)	0.506
Enhanced PRS for type 1 diabetes	0.14 (1.15)	0.05 (1.30)	0.634	0.07 (1.23)	0.737	-0.39 (1.63)	0.461
Enhanced PRS for type 2 diabetes.	0.57 (0.97)	0.62 (0.96)	0.772	0.52 (0.92)	0.684	1.36 (0.91)	0.086

BMI, Body Mass Index; HbA1c, Glycated hemoglobin; ACR, Albumin-to-creatinine ratio; eGFR, Estimated glomerular filtration rate; HDL, High-density lipoprotein; LDL, Low-density lipoprotein; PRS, Polygenic Risk Score.

Kidney Outcomes (Composite): The overarching endpoint including both primary and secondary outcomes. Primary Outcomes (Renal Death): Specifically defined as death directly attributed to renal failure or associated kidney diseases. Secondary Outcomes: Includes clinical endpoints such as End-Stage Renal Disease (ESRD), initiation of Kidney Replacement Therapy (KRT), or a significant decline in eGFR (≥40% decrease from baseline).

Continuous variables are presented as Mean (Standard Deviation [SD]). Differences between groups were analyzed using the independent t-test or the Mann-Whitney U test, depending on the data distribution. Categorical variables are presented as Frequency (Percentage [%]). Differences between groups were evaluated using the Chi-square test or Fisher’s exact test. All P-values are two-sided, with P < 0.05 considered statistically significant.

### Proteomic associations with kidney outcomes

**Figure 1:**
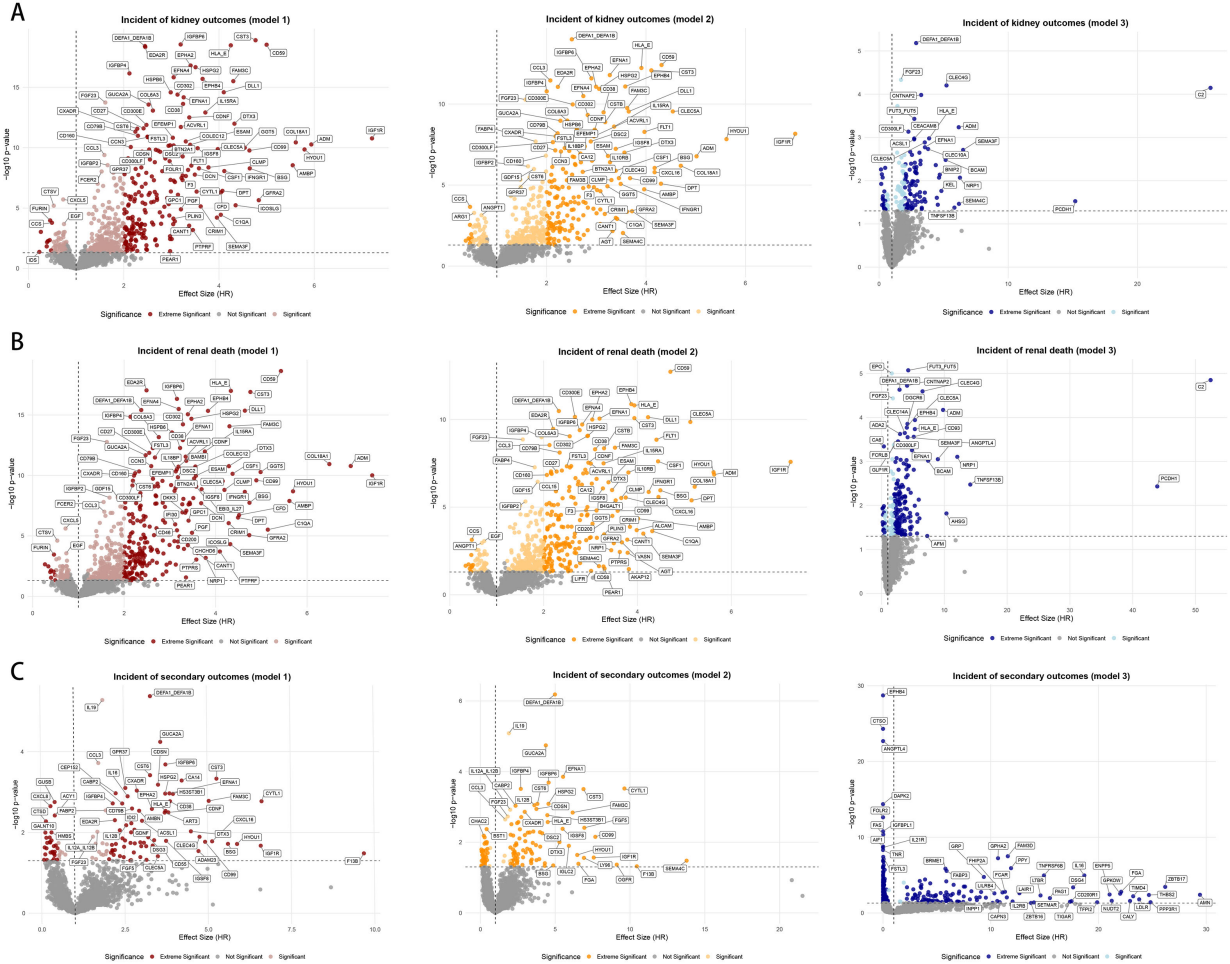
Association of plasma proteins with kidney outcomes in DKD patients. **(A)** Volcano plots displaying the longitudinal association between baseline plasma proteins and the risk of composite kidney outcomes. Model 1 (crude) represents unadjusted analysis. Model 2 (demographic) is adjusted for age, sex, ethnicity, education score, and Townsend deprivation index. Model 3 (fully adjusted) further incorporates metabolic covariates including HbA1c, random glucose, lipid profile [TC, HDL, Lp(a)], urea, BMI, eGFR, UACR, blood pressure, and waist circumference, with a Variance Inflation Factor (VIF) threshold < 10 applied to eliminate collinearity. **(B)** Specific risk associations for the primary outcome (renal death) and **(C)** secondary kidney outcomes. The composite kidney outcome is defined as: (1) renal death (primary outcome); (2) kidney failure (initiation of chronic dialysis, renal transplantation, or sustained eGFR < 15 mL/min/1.73m²); (3) ≥40% decline in eGFR from baseline and (4) ≥30% increase in UACR. DKD, diabetic kidney disease; HR, hazard ratio. Significance categories: “extreme significant” (P < 0.05 and HR > 2.0 or < 0.5) and “significant” (P < 0.05 and HR > 1.2 or < 0.8).

The association patterns between baseline plasma proteins and kidney outcomes across the three Cox regression models was illustrated in **Figure 1**. In the crude model, 561 proteins exhibited significant associations with the composite kidney outcome (HR range 1.64-9.91, all FDR-adjusted P < 0.05). Demographic adjustment retained 508 significant markers (HR range 0.81-9.93). The fully adjusted model incorporating metabolic covariates identified 202 proteins with independent prognostic value (HR range 0.62-9.78). The merge across all three models yielded 633 candidate proteins, including CST3, IGFBP6, CD59, HLA-E, EPHA2, and HSPG2 as the most strongly associated markers (**Figure 1A**). These proteins also maintained significant effects in both the primary (**Figure 1B**) and secondary kidney outcome models (**Figure 1C**).

### Functional enrichment analysis

**Figure 2:**
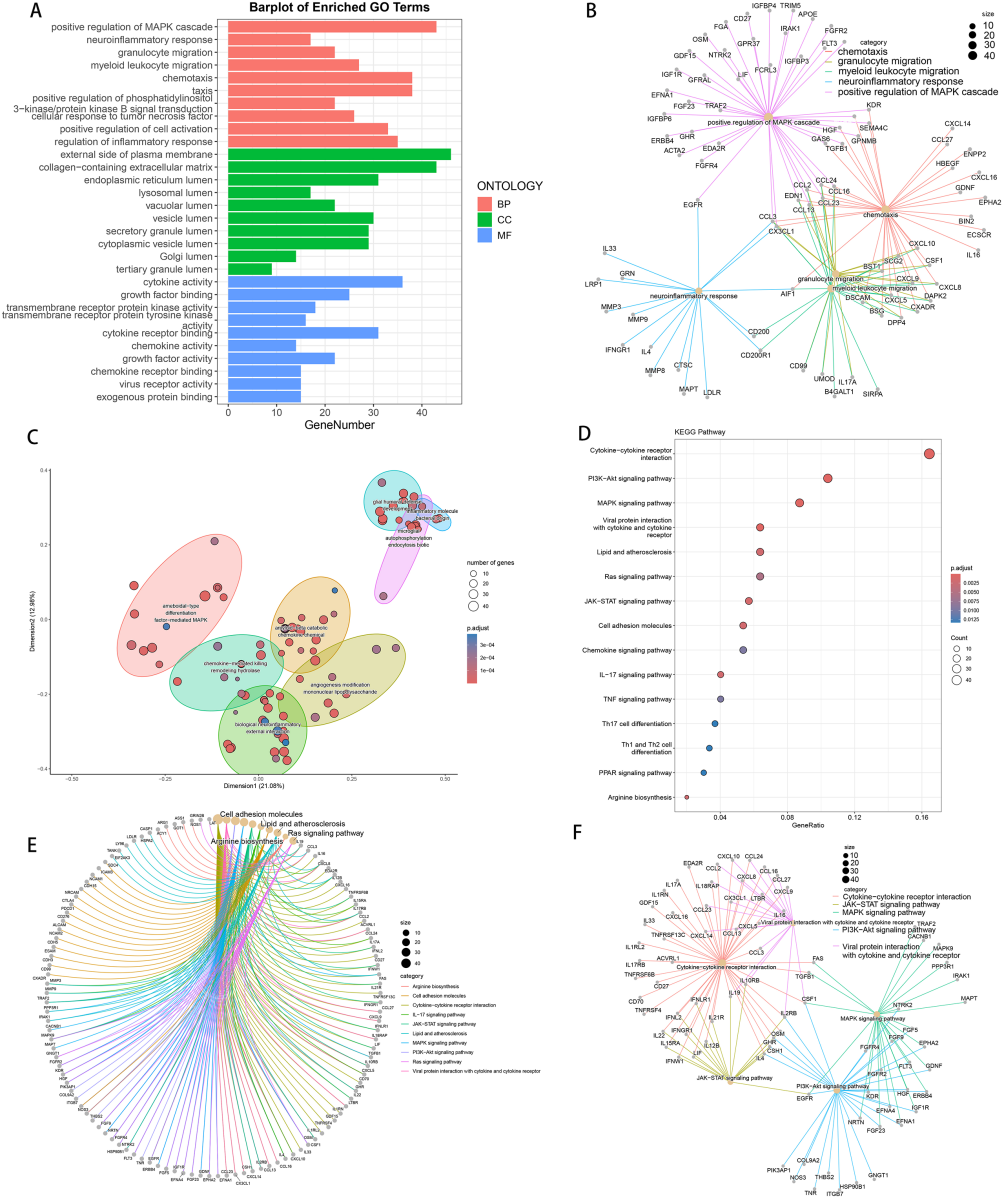
Functional enrichment analysis of proteins associated with kidney disease advancement. **(A–C)** Visualization of Gene Ontology Biological Process (GO-BP) terms reveals a predominance of immunological pathways, including leukocyte chemotaxis, MAPK signaling control, and inflammatory cell recruitment. These clusters demonstrate the systematic coordination of defense mechanisms and transmembrane signaling during disease progression. **(D–F)** KEGG enrichment profiles pinpoint central metabolic and vascular pathways, specifically cytokine interactions and PI3K-Akt signaling. The network diagrams further illustrate intricate crosstalk between major signaling axes and highlight bridge molecules that link extracellular stimuli to intracellular responses in DKD.

The functional enrichment landscape of the 633 proteins was presented in **Figure 2**. Gene Ontology biological process annotation revealed a predominant clustering within immune-inflammatory pathways. Key enriched terms included leukocyte chemotaxis, myeloid cell migration, MAPK cascade regulation, and inflammatory cell recruitment (**Figures 2A–C**). These clusters demonstrate systematic coordination of defense mechanisms and transmembrane signaling during disease progression. Cellular component analysis highlighted membrane raft, lysosomal lumen, and extracellular matrix remodeling as central structural elements. Molecular function enrichment emphasized chemokine binding, glycosaminoglycan interactions, and receptor-ligand activities. KEGG pathway mapping pinpointed cytokine-cytokine receptor interaction, PI3K-Akt signaling, and chemokine signaling as central metabolic and vascular pathways (**Figures 2D–F**). Network visualization illustrated intricate crosstalk between major signaling axes, with bridge molecules linking extracellular stimuli to intracellular responses. These findings collectively implicate sustained immune activation and metabolic dysregulation as fundamental drivers of DKD progression.

### Machine learning feature selection

**Figure 3:**
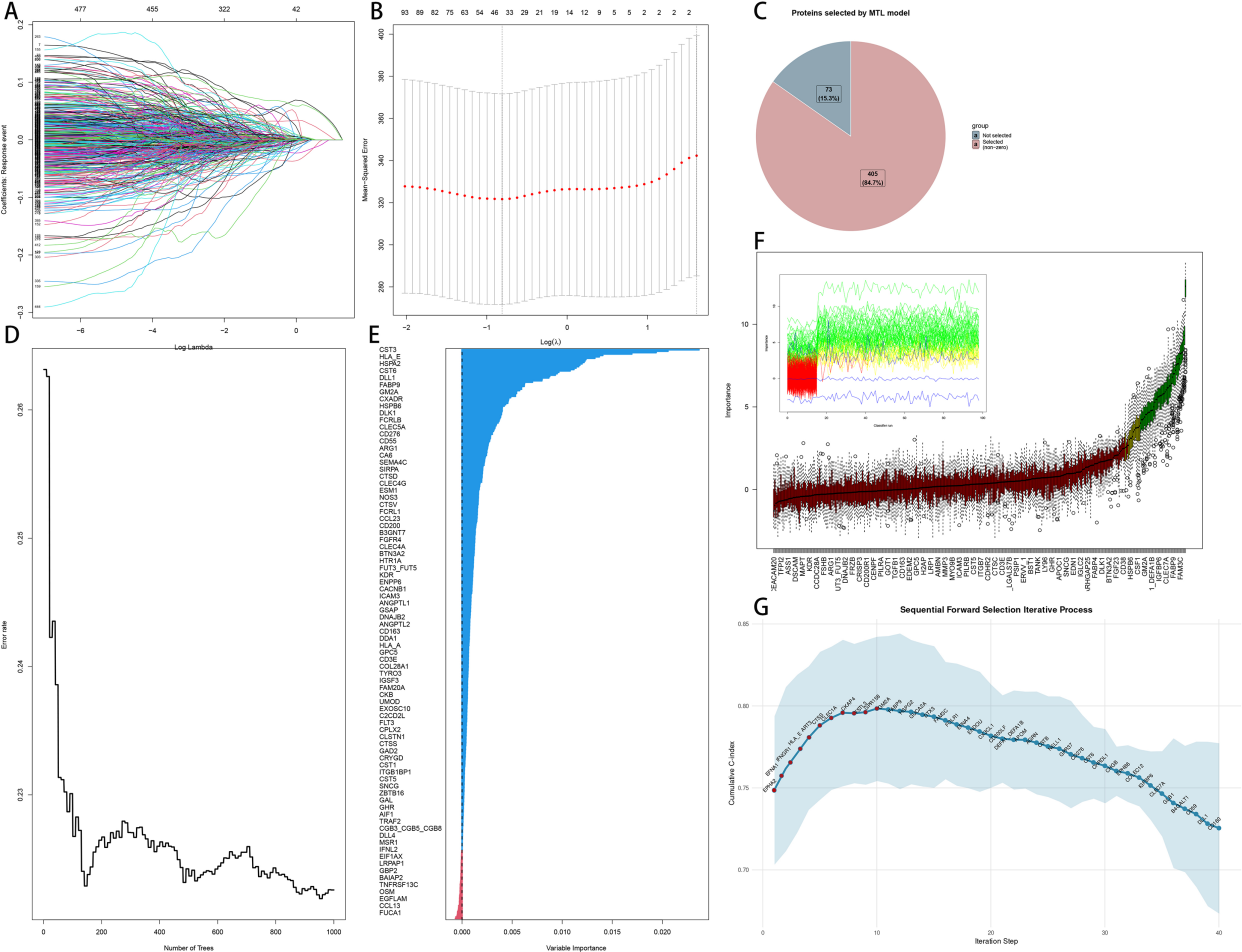
Multistage machine learning framework for the identification of predictive protein signatures. **(A, B)** Feature selection using LASSO-Cox proportional hazards regression. The trajectory of coefficients **(A)** and the selection of the optimal tuning parameter ($\lambda$) via 10-fold cross-validation **(B)** are shown. **(C)** A pie chart illustrating the proportion of selected protein features from the initial candidate pool. **(D, E)** Implementation of Random Survival Forest (RSF). The stabilization of error rates across increasing tree numbers **(D)** and the ranking of protein importance scores **(E)** highlight the top contributors to model performance. **(F)** Boruta all-relevant feature selection. Boxplots represent the Z-scores of candidate proteins compared to shadow features (blue). Confirmed important proteins are shown in green, while rejected and tentative features are in red and yellow, respectively. **(G)** Sequential Forward Selection (SFS) for model parsimony. The cumulative C-index is plotted against the number of variables added, identifying the optimal subset of proteins (in total of 11) where predictive accuracy is maximized.

The multi-stage machine learning framework for protein signature identification was depicted in **Figure 3**. LASSO-Cox regularization (**Figures 3A–C**) applied stringent selection criteria, retaining proteins with non-zero coefficients in ≥50% of cross-validation folds. This procedure yielded 92 candidate predictors from the initial 633-protein pool. The trajectory of coefficients demonstrated progressive shrinkage as λ increased, with optimal parameter selection via minimum cross-validated error plus one standard error. Random Survival Forest analysis (**Figures 3D, E**) evaluated importance scores for LASSO-retained proteins. Error rates stabilized beyond 500 trees, confirming model convergence. The majority of proteins demonstrated positive permutation importance, indicating genuine predictive contribution rather than random noise. The Boruta algorithm (**Figure 3F**) confirmed 33 proteins as all-relevant features after 100 iterations. Confirmed important proteins (green) exhibited Z-scores consistently exceeding shadow feature baselines, while rejected (red) and tentative (yellow) features were excluded from downstream analysis. Sequential Forward Selection (**Figure 3G**) tracked cumulative C-index as variables were iteratively incorporated. Predictive performance increased monotonically until reaching a plateau at 11 proteins, beyond which additional variables contributed minimal incremental value. This procedure identified the optimal parsimonious subset: HLA-E, EFNA1, GPR158, FSTL3, ART3, GM2A, CLEC1A, CKAP4, IFNGR1, CTSS, and EPHA2.

### XGBoost and SHAP interpretability analysis

**Figure 4:**
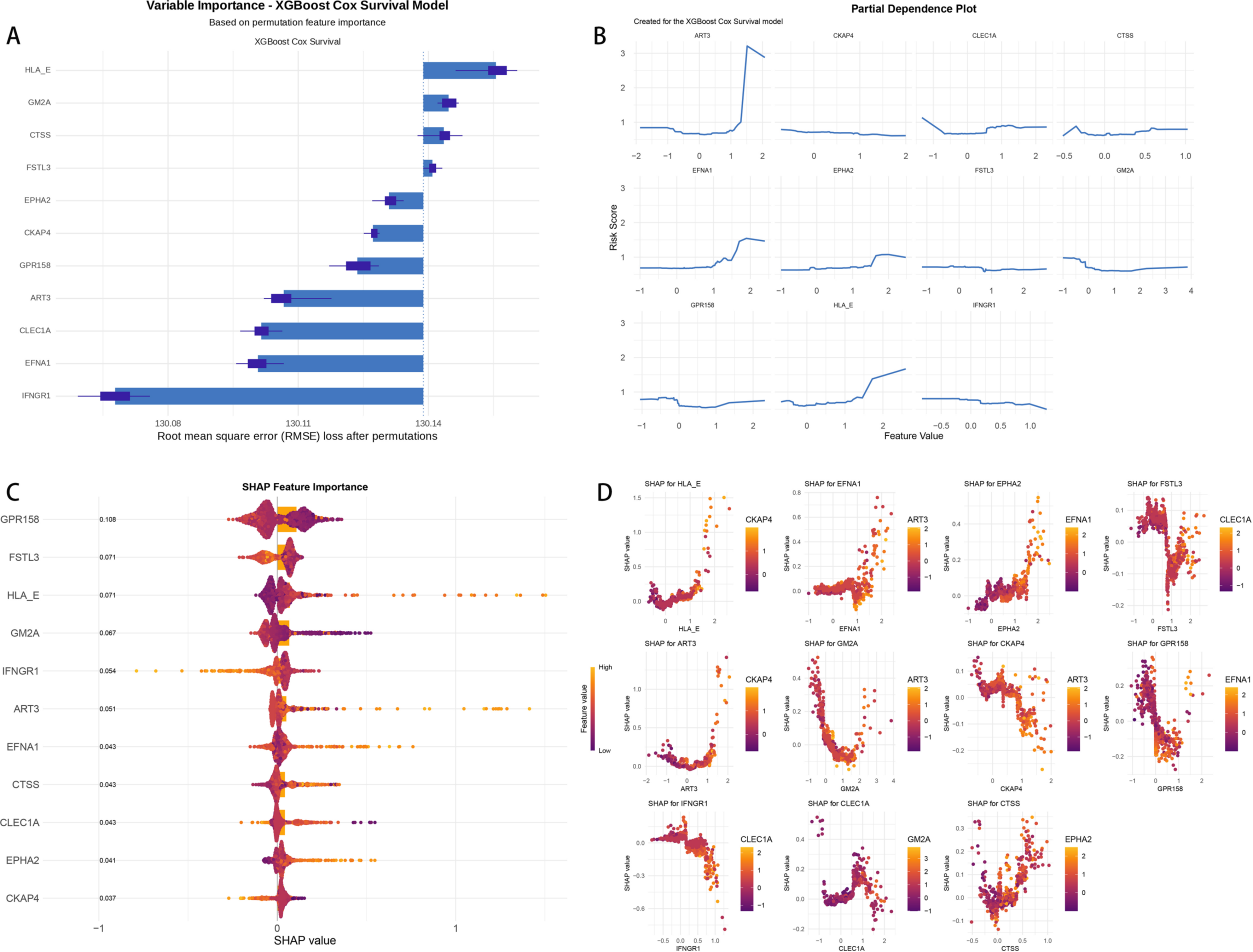
Machine learning-based interpretability analysis of the final protein signature. **(A)** Variable Importance via Permutation. The bar chart ranks the top 11 proteins based on the Root Mean Square Error (RMSE) loss after feature permutation in the XGBoost Cox Survival model. Higher loss values indicate a greater contribution of the protein (e.g., HLA-E, GM2A, CTSS) to the model’s predictive accuracy. **(B)** Partial Dependence Plots (PDP). These plots visualize the marginal effect of individual proteins on the predicted risk score. Most proteins exhibit a non-linear relationship, where risk scores sharply transition or plateau at specific feature concentration thresholds (e.g., ART3, EFNA1, and HLA-E show distinct risk escalations as their levels increase). **(C)** SHAP Feature Importance. The SHAP (SHapley Additive exPlanations) summary plot illustrates the distribution of the impact each protein has on the model output. Each dot represents a patient; the color indicates the feature value (red for high, purple for low). For instance, high levels of GPR158 and HLA-E are associated with increased SHAP values, indicating higher predicted risk. **(D)** SHAP Interaction Plots. These scatter plots reveal how the impact of one protein (SHAP value on the y-axis) is conditioned by its own value and the concentration of a second interacting protein (color-coded). This highlights the complex, synergistic biological interactions between protein pairs (e.g., HLA-E and CKAP4, IFNGR1 and CLEC1A) in driving kidney disease progression.

The machine learning-based interpretability analysis of the 11-protein signature was presented in **Figure 4**. Variable importance via permutation ranking (**Figure 4A**) identified HLA-E, GM2A, and CTSS as the top contributors to model performance, with feature permutation causing substantial predictive degradation. Partial Dependence Plots (**Figure 4B**) visualized marginal effects of individual proteins on predicted risk scores. Most proteins exhibited non-linear relationships, with risk scores transitioning sharply at specific concentration thresholds. Notably, ART3, EFNA1, and HLA-E demonstrated distinct risk escalations as their levels increased.

SHAP feature importance (**Figure 4C**) illustrated the distribution of impact each protein exerted on model output. High levels of GPR158 and HLA-E were associated with increased SHAP values, indicating elevated predicted risk. SHAP interaction plots (**Figure 4D**) revealed synergistic relationships between protein pairs, with the impact of one protein conditioned by concentrations of interacting partners. HLA-E demonstrated particularly strong interaction effects with CKAP4, while IFNGR1 and CLEC1A exhibited coordinated risk amplification.

### Correlation with clinical variables

**Supplementary Figure 3** presents correlation analysis between the 11 candidate proteins and clinical indicators. The correlation matrix revealed strong positive associations between most proteins and renal injury markers (cystatin C, urea), alongside significant negative correlations with kidney function indicators (eGFR). Individual linear regression plots demonstrated highly significant linear relationships between eGFR and protein levels for HLA-E, EFNA1, EPHA2, FSTL3, ART3, GM2A, CLEC1A, CKAP4, GPR158, and IFNGR1 (all P < 0.001), with Pearson correlation coefficients typically ranging from -0.4 to -0.6. CTSS showed attenuated correlation with eGFR, foreshadowing its subsequent exclusion from the final signature.

### Survival analysis and protein validation

**Figure 5:**
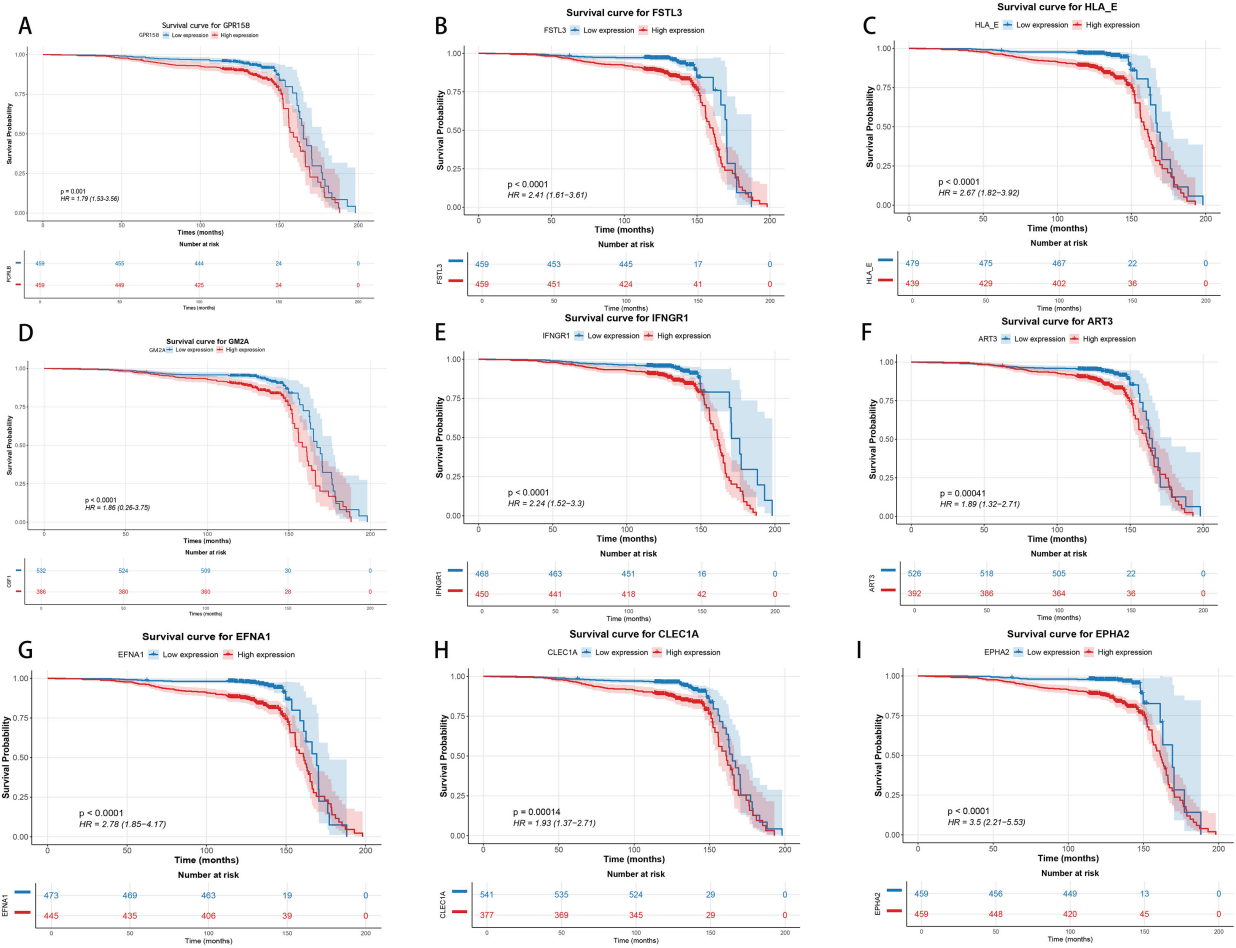
Kaplan-Meier survival analysis for the identified key proteins in DKD progression. **(A–I)** Kaplan-Meier curves illustrate the cumulative probability of event-free survival for the top-ranked proteins, including GPR158 **(A)**, FSTL3 **(B)**, HLA-E **(C)**, GM2A **(D)**, IFNGR1 **(E)**, ART3 **(F)**, EFNA1 **(G)**, CLEC1A **(H)**, and EPHA2 **(I)**. Patients were stratified into high-expression (red line) and low-expression (blue line) groups based on the median concentration of each protein. For nearly all identified biomarkers (e.g., HLA-E, EFNA1, and EPHA2), high baseline plasma levels are significantly associated with a decreased survival probability and an increased risk of incident kidney outcomes (Log-rank P < 0.001). The Hazard Ratios (HR) and 95% Confidence Intervals (CI) highlight the robust predictive strength of these individual proteins, with EPHA2 (HR = 3.50, 95%CI:2.21-5.53) and EFNA1 (HR = 2.78, 95%CI:1.85-4.17) showing particularly strong associations with adverse prognosis.

Kaplan-Meier survival curves for the identified proteins were displayed in **Figure 5**. Patients were stratified into high-expression (above median) and low-expression (below median) groups based on baseline protein concentrations. For nine of eleven proteins, GPR158 (**Figure 5A**), FSTL3 (**Figure 5B**), HLA-E (**Figure 5C**), GM2A (**Figure 5D**), IFNGR1 (**Figure 5E**), ART3 (**Figure 5F**), EFNA1 (**Figure 5G**), CLEC1A (**Figure 5H**), and EPHA2 (**Figure 5I**), high baseline plasma levels were significantly associated with decreased event-free survival and increased risk of incident kidney outcomes (all Log-rank P < 0.001).

Hazard ratios quantified the prognostic strength of individual proteins. EPHA2 demonstrated the strongest association (HR = 3.50, 95% CI 2.21-5.53), followed by EFNA1 (HR = 2.78, 95% CI 1.85-4.17), HLA-E (HR = 2.67, 95% CI 1.82-3.92), and GPR158 (HR = 1.79, 95% CI 1.53-3.56). These findings validate the machine learning selection by demonstrating independent risk stratification capability.

**Supplementary Figure 4** presents survival and trajectory analyses for CTSS and CKAP4. Notably, CTSS showed no significant survival separation (P = 0.766) and no trajectory divergence between cases and controls (P = 0.748). In contrast, CKAP4 demonstrated significant survival association (P = 0.00065) and marked trajectory divergence (P = 1.84×10^-8^). Based on these findings, CTSS was excluded from the final signature, yielding a refined 10-protein panel.

### Longitudinal trajectory analysis

**Figure 6:**
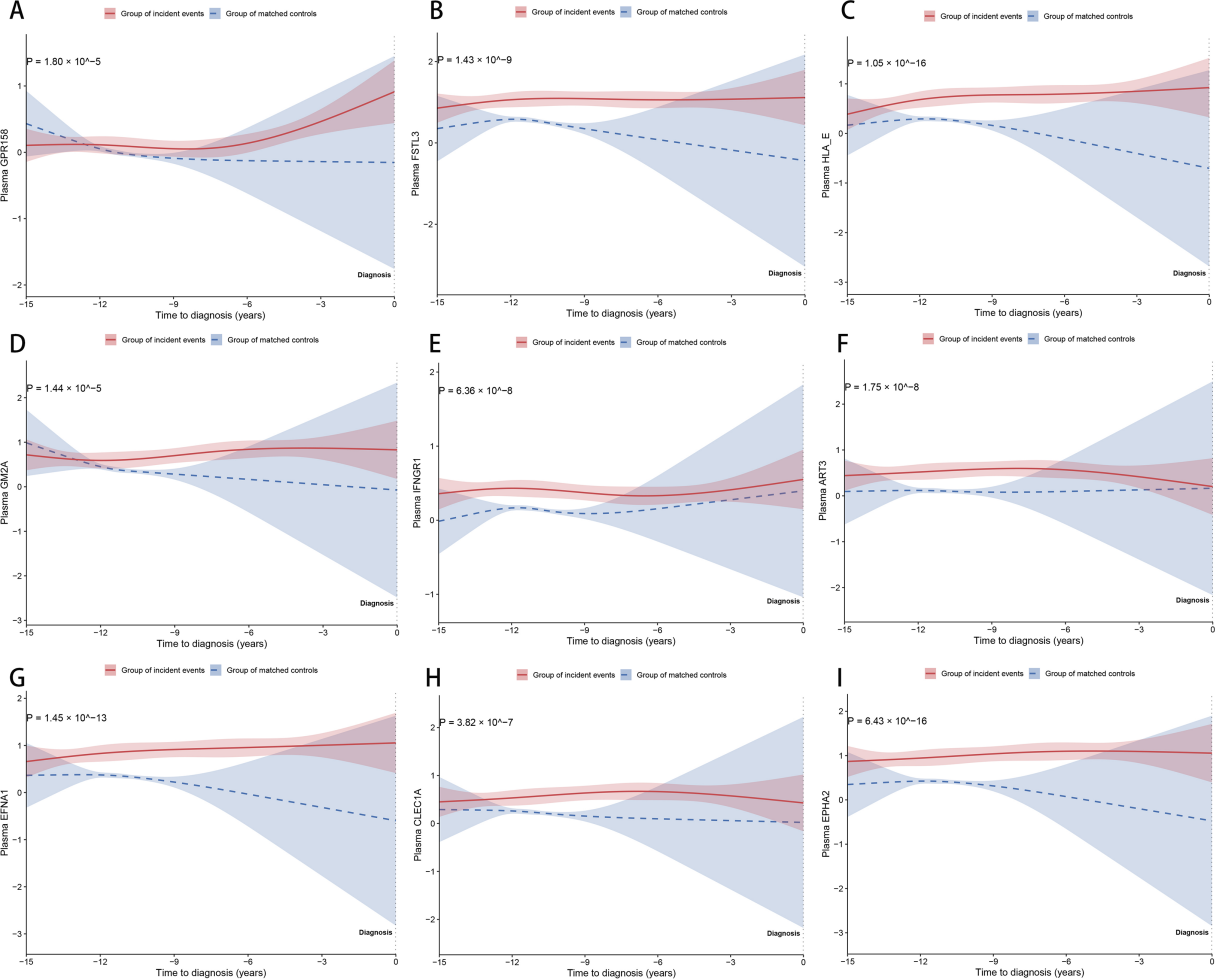
Longitudinal trajectories of key plasma proteins prior to the diagnosis of incident kidney outcomes. **(A–I)** Trajectory plots illustrate the dynamic changes in standardized plasma levels for GPR158 **(A)**, FSTL3 **(B)**, HLA-E **(C)**, GM2A **(D)**, IFNGR1 **(E)**, ART3 **(F)**, EFNA1 **(G)**, CLEC1A **(H)**, and EPHA2 **(I)** over a 15-year period preceding the diagnosis. The red solid lines represent the group with incident kidney events, while the blue dashed lines represent the age- and sex-matched control group. Shaded areas indicate the 95% confidence intervals. For most identified biomarkers, such as HLA-E **(C)**, EFNA1 **(G)**, and EPHA2 **(I)**, a significant divergence between the two groups is observed years before the clinical diagnosis (P-values ranging from 1.80 x 10^ -5 to 1.05 x 10^ -16). These proteins typically show a steady upward trend in the incident event group as the time to diagnosis approaches, underscoring their potential for early risk stratification and pre-clinical monitoring.

15-year pre-diagnostic trajectories for the 10 validated proteins were illustrated in **Figure 6** and **Supplementary Figure 4**. In the analysis, incident cases (red solid lines) and matched controls (blue dashed lines) were tracked from 15 years before diagnosis. For HLA-E (**Figure 6C**), EFNA1 (**Figure 6G**), EPHA2 (**Figure 6I**), and most other proteins, significant divergence between groups emerged years before clinical diagnosis, with cases demonstrating progressive elevation as the endpoint approached. Specifically, GPR158 (**Figure 6A**) and GM2A (**Figure 6D**) exhibited modest early separation with gradual divergence, while FSTL3 (**Figure 6B**) and ART3 (**Figure 6F**) maintained relatively stable elevated levels throughout the pre-diagnostic period. In contrast, IFNGR1 (**Figure 6E**) and CLEC1A (**Figure 6H**) showed more pronounced divergence only in the later years approaching diagnosis. These distinct temporal patterns suggest heterogeneous biological processes underlying kidney disease progression, with certain proteins serving as early warning indicators and others reflecting advanced pathological changes. Mann-Kendall trend test P-values ranged from 1.80×10–^5^ to 1.05×10^-16^, underscoring the statistical robustness of trajectory divergence.

### Predictive performance benchmarking

**Figure 7:**
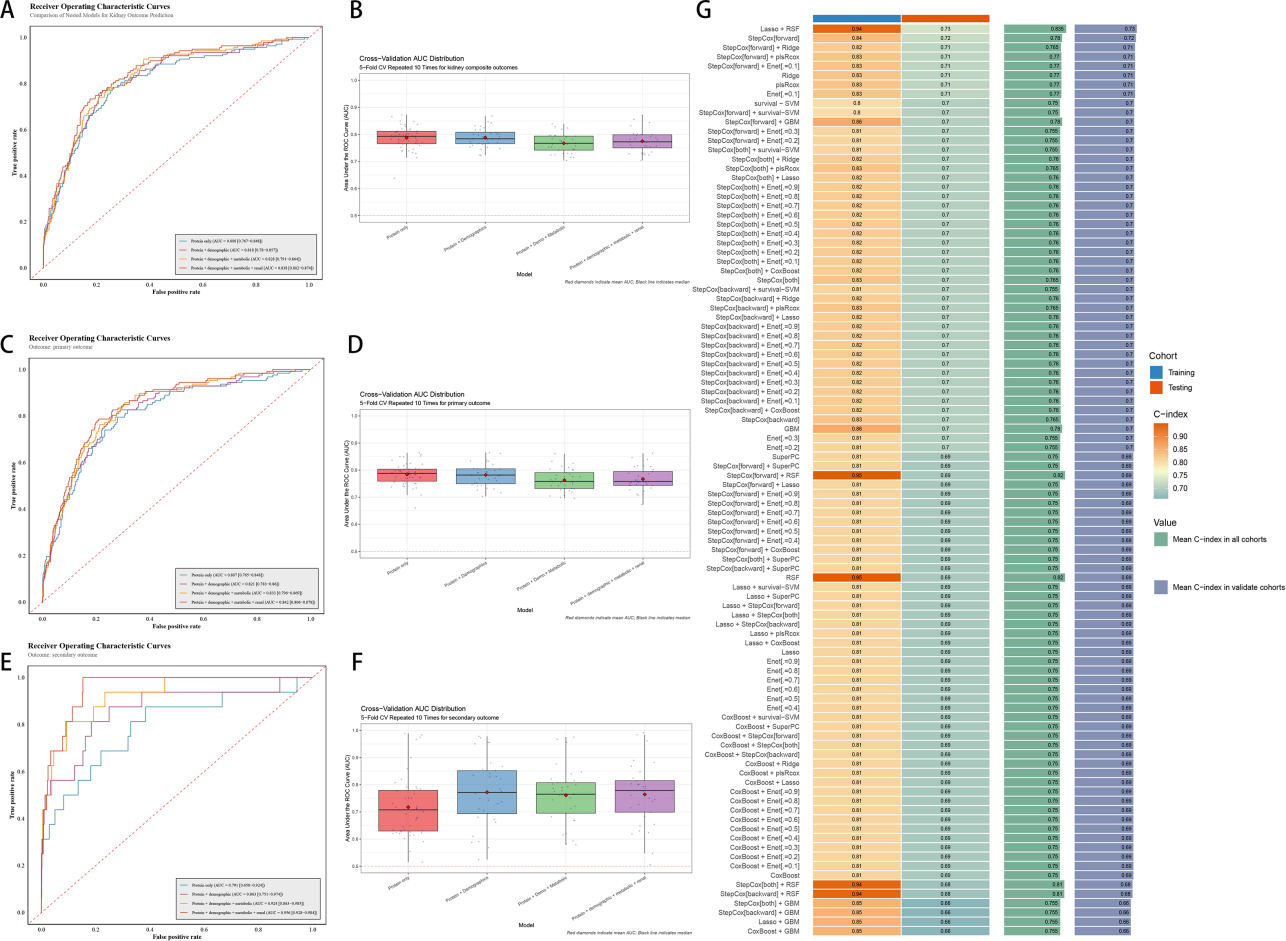
Performance evaluation and benchmarking of the validated 10-protein signature. The analysis was based on a core 10-protein signature, established after excluding CTSS due to its non-significant survival separation (P > 0.05) in Kaplan-Meier analysis. **(A–F)** Predictive accuracy across multi-component models. ROC curves and AUC distributions are presented for the composite kidney outcome **(A, B)**, renal death **(C, D)**, and secondary endpoints **(E, F)**. Four models were compared: (1) Protein-only; (2) Protein + Demographics; (3) Protein + Metabolic traits; and (4) Protein + Demographics + Metabolic traits. The Protein-only model achieved high predictive accuracy, showing nearly identical performance to the more complex integrated models. Notably, in the repeated cross-validation analysis **(B, D)**, the Protein-only model demonstrated even higher and more stable AUC values, reinforcing its superior generalizability. **(G)** Systematic benchmarking of machine learning algorithms via Mime1. To evaluate the robustness of the 10-protein signature, the original cohort was split into a Training set (70%) and a Testing set (30%). Among 101 algorithm combinations screened, the Lasso + RSF (Random Survival Forest) framework was selected as the final optimal model due to its exceptional stability and high C-index performance across both training (orange) and independent testing (blue) cohorts.

Comprehensive performance evaluation of the validated 10-protein signature was presented in **Figure 7**. ROC analyses compared four sequential modeling approaches across composite kidney outcomes (**Figures 7A, B**), renal death (**Figures 7C, D**), and secondary endpoints (**Figures 7E, F**). The protein-only model achieved AUC = 0.808[0.767-0.848] for composite outcomes and AUC = 0.807[0.765-0.848] for renal death, performance nearly identical to more complex integrated models incorporating demographics and metabolic traits.

Repeated cross-validation (**Figures 7B, D**) demonstrated even higher and more stable AUC values for the protein-only approach, reinforcing its superior generalizability. DeLong tests found no statistically significant differences between protein-only and integrated models (all P > 0.05), establishing that the 10-protein signature captures the essential prognostic information without requiring extensive clinical covariates.

Systematic algorithm benchmarking via 101 method combinations (**Figure 7G**) identified LASSO-RSF as the optimal framework, achieving C-index = 0.94 in the training cohort and 0.73 in the independent testing cohort. This performance exceeded alternative approaches including naive Bayes, support vector machines, and k-nearest neighbors, validating the analytical pipeline selection.

To further rigorously evaluate the stability of our full analytical pipeline and mitigate the risk of overfitting, we conducted a 10-fold nested internal cross-validation comparing LASSO+RSF against five alternative survival algorithms (**Supplementary Figure 5A**). The LASSO+RSF framework consistently demonstrated superior robustness, exhibiting both the highest median C-index and the narrowest interquartile range across all folds. Specifically, LASSO+RSF achieved a mean C-index of 0.884, significantly surpassing standalone RSF (0.842) and penalized Cox models (**Supplementary Figure 5B**).

Model calibration was systematically assessed at three critical follow-up milestones (**Supplementary Figure 5C**). The Integrated Calibration Index (ICI) was 0.0194 at the first quartile (Q1: 73 months) and 0.0221 at the median (127 months), indicating high reliability and precise risk estimation during the early-to-mid follow-up periods. At the long-term horizon (Q3: 157 months), we observed an increased variance in calibration (ICI = 0.1797), likely reflecting the inherent statistical challenges of sparse data at the survival curve’s tail. However, the calibration slope of 1.198 and intercept of 0.198 at this stage suggest the model remains reasonably balanced without exhibiting significant systematic bias.

Sensitivity analysis restricted to the 408 participants meeting the KDIGO laboratory-based definition of DKD, among whom 106 experienced the composite kidney outcome, confirmed the robustness of the 10-protein signature. The protein-only model retained high discriminative capacity with an area under the receiver operating characteristic curve (AUC) of 0.749 (95% CI 0.694-0.804). In repeated 10-fold cross-validation (50 iterations), the protein-only model yielded consistently higher and more stable AUC distributions compared with models incorporating demographic or metabolic covariates (**Supplementary Figure 6**). These results collectively confirm that the 10-protein signature provided a stable and well-calibrated tool for longitudinal risk stratification.

### Web application deployment

**Figure 8:**
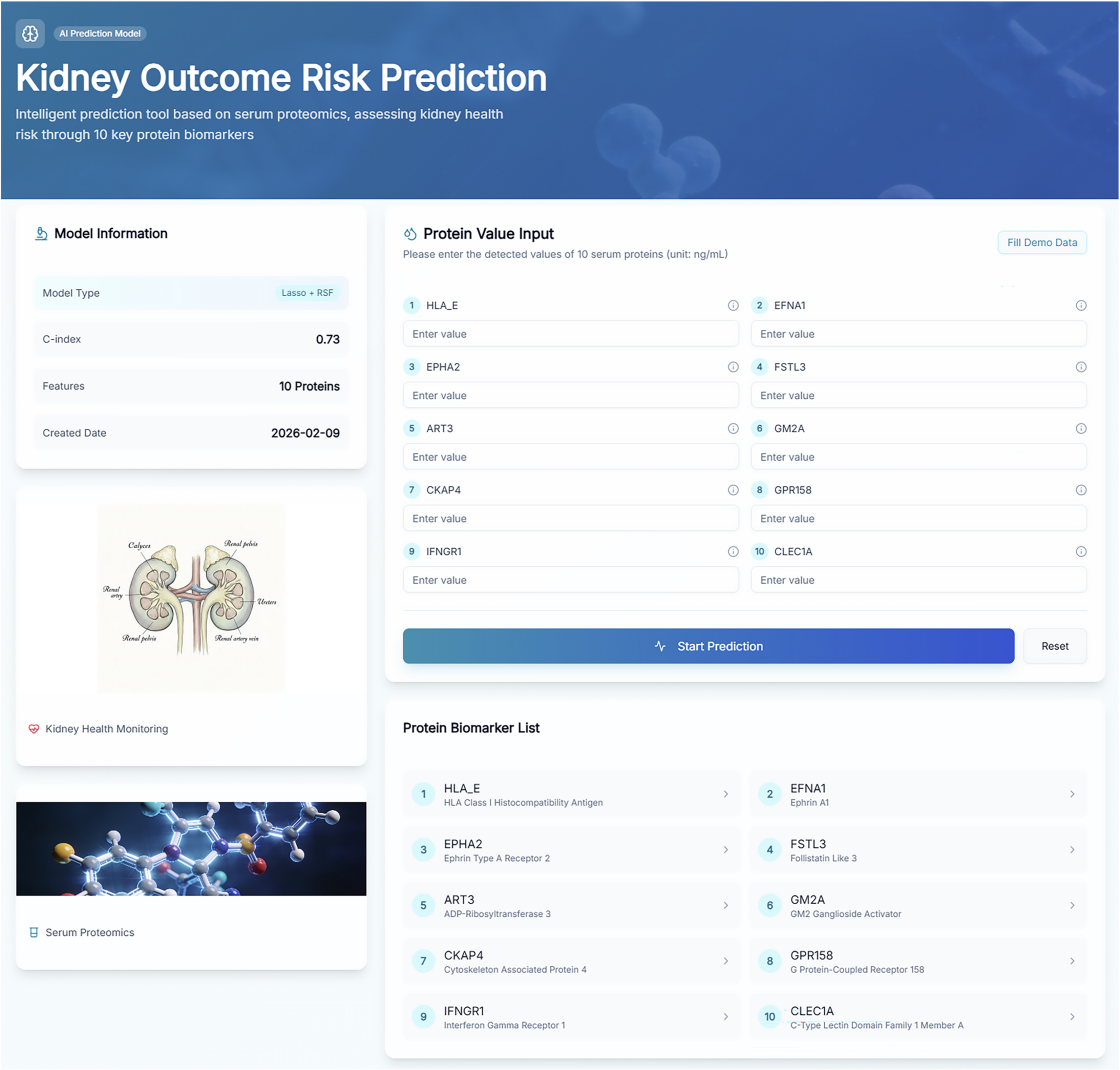
Web-based application for kidney outcome risk prediction in DKD patients. To facilitate clinical translation, the optimized Lasso + RSF model was deployed as a user-friendly, interactive web tool (available at: https://jiangli2941.github.io/kidney-outcomes-prediction/). The interface comprises three functional modules: (1) Model Information, which displays the core parameters, including the algorithm type, a C-index of 0.73, and the utilization of the 10 key protein biomarkers; (2) Protein Value Input, allowing clinicians to enter quantitative serum proteomics data (NPX) for individual patients across the 10 validated markers (e.g., HLA-E, EFNA1, GPR158); and (3) Biomarker Reference List, providing detailed biological annotations for each protein in the signature. This digital platform provides an intelligent, evidence-based assessment of kidney health risks, enabling personalized prognosis monitoring for individuals with diabetic kidney disease.

The deployed web-based prediction tool was illustrated in **Figure 8**. The interface enables clinicians to input patient-level protein concentrations for the 10 validated biomarkers, with real-time calculation of kidney outcome risk probability. The application displays model parameters, protein reference ranges, and biomarker annotations to support clinical interpretation. This digital platform translates the research findings into an accessible, evidence-based decision support system for personalized DKD management.

## Discussion

In this prospective cohort investigation integrating large-scale plasma proteomics with multi-stage machine learning, we identified and validated a 10-protein signature capable of predicting kidney outcomes in patients with diabetic kidney disease. Our findings demonstrate that plasma proteomic profiling, when combined with rigorous analytical frameworks, can distill robust prognostic biomarkers that capture pre-clinical disease processes and enable early risk stratification. The deployable web application also facilitates clinical translation.

The 633 proteins associated with kidney outcomes across sequential Cox models implicate immune-inflammatory pathways and metabolic signaling cascades as central to DKD progression. Gene Ontology enrichment revealed leukocyte chemotaxis, MAPK cascade regulation, and inflammatory cell recruitment as predominant biological processes. These findings align with contemporary understanding of DKD pathophysiology, wherein sustained low-grade inflammation, driven by hyperglycemia, advanced glycation end-products, and hemodynamic stress, propagates glomerular and tubulointerstitial injury (32–34).

Machine learning selection identified 10 core proteins with validated prognostic utility. HLA-E, a non-classical major histocompatibility complex class I molecule, emerged as a top-ranked predictor. Beyond its canonical role in immune surveillance, HLA-E has been implicated in vascular inflammation and endothelial activation (35). Elevated circulating HLA-E may reflect immune-mediated renal injury or systemic inflammation propagating across vascular beds (36).

EFNA1 and its receptor EPHA2, both identified in our signature, mediate bidirectional signaling regulating cell adhesion, migration, and vascular remodeling. The ephrin-Eph system modulates glomerular permeability and podocyte integrity. Dysregulated signaling through this system also contributes to proteinuria and progressive renal damage (37, 38). Our finding that both ligand and receptor predict kidney outcomes suggests coordinated pathway activation in DKD pathogenesis.

GPR158, an orphan G-protein coupled receptor highly expressed in neural and renal tissues, represents a novel DKD biomarker. Emerging evidence implicates GPR158 in metabolic regulation and stress responses (39). Its elevation in progressive DKD may reflect adaptive or maladaptive responses to chronic hyperglycemia and oxidative stress (40).The 10-protein signature captured three interconnected DKD mechanisms: endothelial dysfunction (EPHA2-EFNA1 axis compromising glomerular barrier, CLEC1A altering vascular homeostasis) (41), inflammation (CLEC1A facilitating leukocyte recruitment) (42), and fibrosis (FSTL3 reflecting failed TGF-β inhibition, CKAP4 marking podocyte cytoskeletal injury) (43, 44). These proteins established a feed-forward cascade where metabolic stress initiates endothelial injury, inflammatory amplification ensues, and sustained TGF-β signaling drives irreversible scarring.

The finding that a protein-only predictive model achieved performance comparable to integrated models incorporating extensive demographic and metabolic covariates carries important clinical implications. Current risk stratification tools for DKD require comprehensive clinical data collection, limiting their utility in resource-constrained settings. A blood-based protein panel offers several advantages: objective quantification, biological specificity for disease processes, and potential for high-throughput automation. Our ROC analyses demonstrate that the 10-protein signature alone achieves AUC > 0.80 for both composite outcomes and renal death, discriminative capacity sufficient for clinical utility.

Longitudinal trajectory analysis revealed that protein divergence between future cases and controls emerges years before clinical diagnosis. This temporal pattern establishes that the identified biomarkers capture pre-clinical pathophysiological processes rather than merely reflecting established damage. The progressive elevation of proteins like HLA-E, EFNA1, and EPHA2 as the endpoint approaches suggests dynamic disease activity that could be monitored to guide intervention timing and intensity.

Several limitations warrant consideration. First, the model lack validation in a truly independent external cohort. Currently, both the development and testing of the 10-protein signature were conducted among participants from the UK Biobank. Although this represents a large and diverse population, the study participants are predominantly of European descent and were recruited under a specific healthcare system, which may introduce healthy-volunteer bias. Consequently, our web tool could only provide a preliminary exploration result. Second, proteomic measurements were obtained at a single baseline timepoint. The dynamic changes in protein levels over time may provide additional prognostic information. Serial sampling studies could elucidate optimal monitoring intervals and trajectory patterns predictive of imminent risk. Third, while we identified robust statistical associations, the observational design precludes definitive causal inference.

Future research would validate the signature in independent, ethnically diverse cohorts, compare it head-to-head with established risk scores, assess its incremental value when added to clinical variables, and investigate protein changes following therapeutic interventions. Ultimately, prospective trials are needed to determine whether risk stratification using the 10-protein signature improves patient outcomes through intensified monitoring and preemptive therapy.

In conclusion, this study establishes a validated plasma proteomic signature for early prediction of kidney outcomes in diabetic kidney disease. The identified biomarkers illuminate immune-inflammatory and metabolic pathways driving disease progression while providing a foundation for precision nephrology. The machine learning framework and web-based tool offer accessible, interpretable risk assessment to support clinical decision-making and optimize patient care.

## Data Availability

The original contributions presented in the study are included in the article/**Supplementary Material**. Further inquiries can be directed to the corresponding authors.
