# Supplementary material for: Plasma proteomics based on machine learning predict the early risk of kidney outcomes in patients with DKD: a prospective cohort study from UK Biobank
Source: Front Nephrol. 2026 Jun 29;6:1862772. doi: 10.3389/fneph.2026.1862772 (PMC13357851; doi:10.3389/fneph.2026.1862772)
Supplement: Supplementary file 1 [file DataSheet1.pdf]

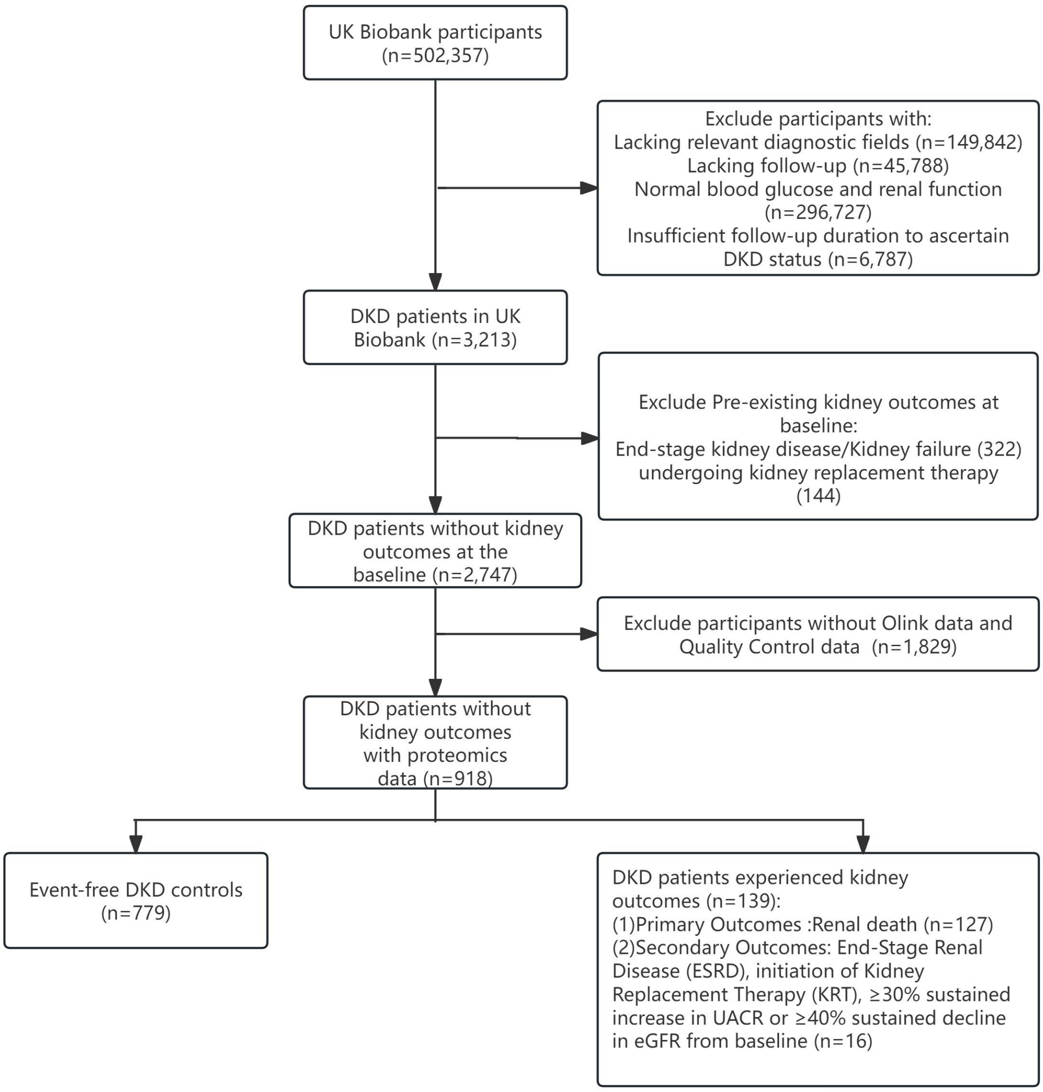

Supplementary Fig. 1 Study flow diagram of participant selection and clinical outcomes.
Starting from the UK Biobank cohort (n=502,357), participants were excluded for lacking relevant diagnostic fields, follow-up data, or normal baseline renal function, resulting in 3,213 individuals with Diabetic Kidney Disease (DKD). We further excluded those with pre-existing kidney failure or undergoing replacement therapy at baseline. After filtering for available proteomic and quality control data, a final study population of 918 patients was identified. During the follow-up period, 779 patients remained event-free (controls), while 139 patients experienced incident composite kidney outcomes. The incident events comprised 127 primary outcomes (renal death) and 16 secondary outcomes (including ESRD, kidney replacement therapy, ≥30% sustained increase in UACR or ≥40% sustained decline in eGFR from baseline).

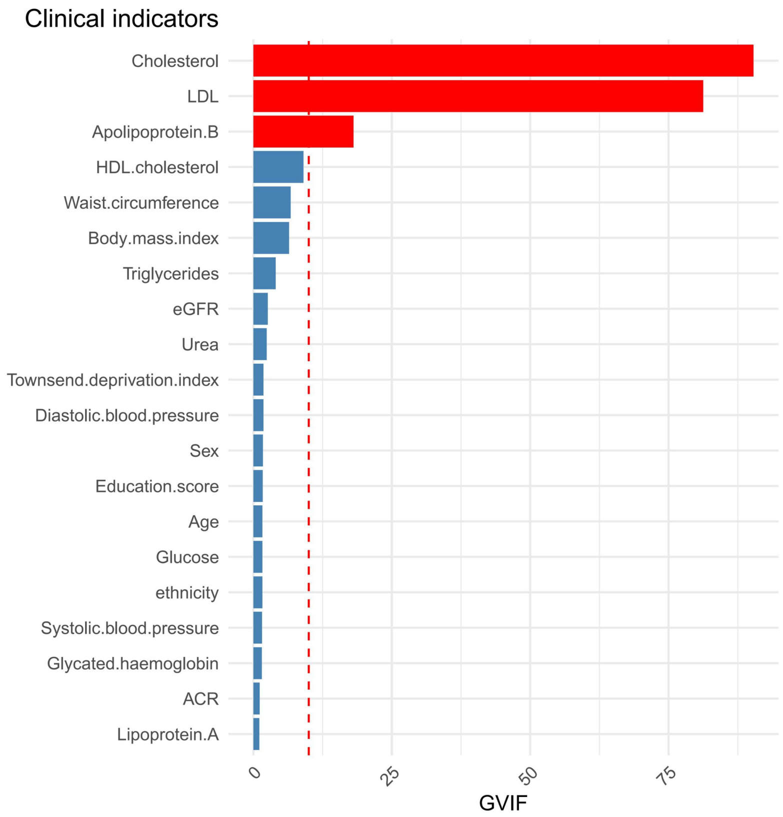

Supplementary Fig. 2 Collinearity assessment of clinical and metabolic covariates via Generalized Variance Inflation Factor (GVIF).

The bar chart illustrates the GVIF values for the metabolic and clinical covariates included in the fully adjusted Model 3. To ensure model stability and mitigate multicollinearity, a stringent GVIF threshold of 10 was applied (indicated by the red dashed line). Covariates exceeding this threshold, specifically Cholesterol, LDL, and Apolipoprotein B, are highlighted in red, indicating high collinearity with other lipid parameters. These redundant features were subsequently eliminated from the multivariate analysis. In contrast, essential predictors such as eGFR, ACR, HbA1c, and systolic blood pressure exhibited GVIF values well below the threshold (shown in steel blue), confirming their independence and suitability for inclusion in the final Cox proportional hazards models.

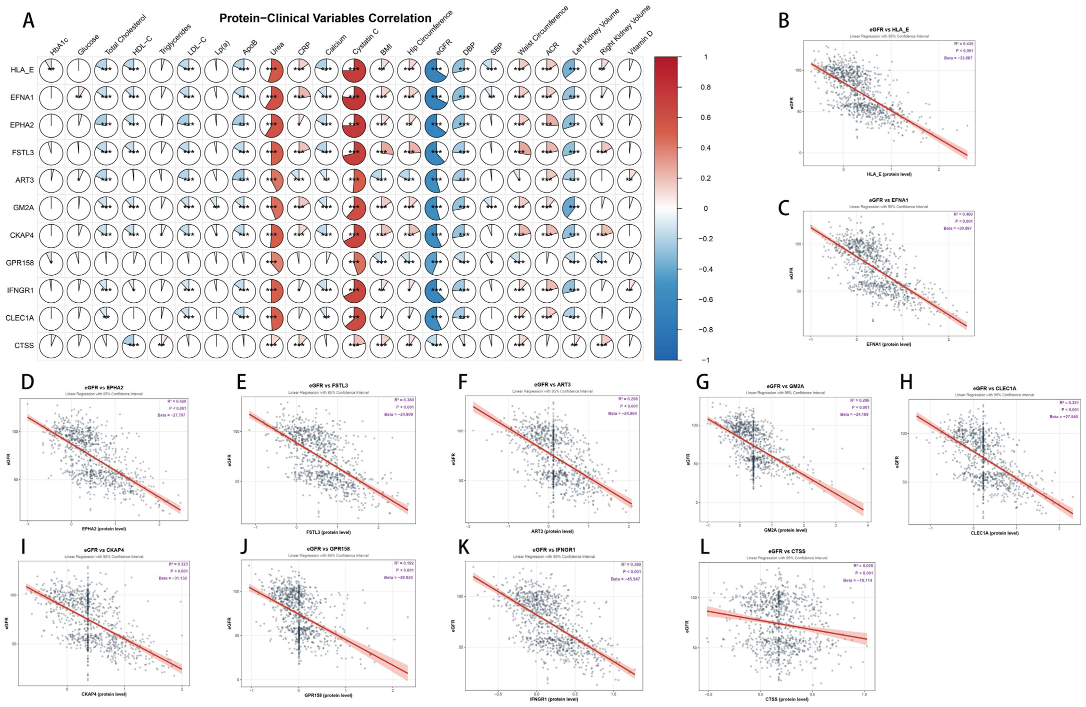

Supplementary Fig. 3 Correlation analysis between hub proteins and clinical variables.

(A) Matrix of correlations. The corrplot summarizes the Pearson correlation coefficients between the 12 candidate proteins (including HLA-E, EFNA1, GPR158, etc.) and various clinical indicators (e.g., eGFR, Cystatin C, Urea, ACR). The color and fill level of the circles represent the strength and direction of the correlation (red for positive, blue for negative). Most identified proteins show strong positive correlations with renal injury markers (Cystatin C and Urea) and significant negative correlations with kidney function markers (eGFR). (B – L) Individual linear regression plots. Scatter plots with regression lines illustrate the specific quantitative relationship between eGFR and individual proteins: HLA-E (B), EFNA1 (C), EPHA2 (D), FSTL3 (E), ART3 (F), GM2A (G), CLEC1A (H), CKAP4 (I), GPR158 (J), IFNGR1 (K), and CTSS (L). All proteins, except for CTSS, demonstrate a highly significant linear decline in eGFR as protein levels increase (P < 0.001), with Pearson correlation coefficients (R) typically ranging from -0.4 to -0.6, underscoring their strong association with diminished renal function.

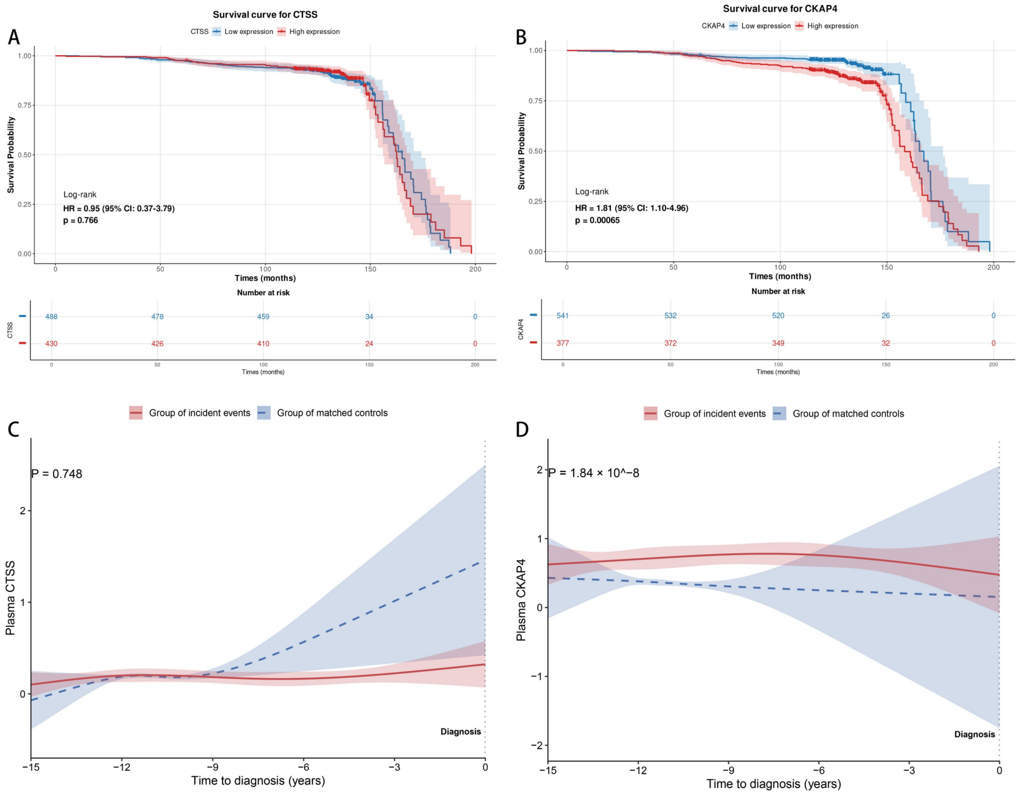

Supplementary Fig. 4 Kaplan-Meier Survival Curves and trajectory based on the expression levels of CTSS (A) and CKAP4 (B). For CTSS, there is no statistically significant difference in survival between high (red) and low (blue) expression groups (p = 0.766). Conversely, for CKAP4, high expression is significantly associated with poorer survival outcomes (HR = 1.81, p = 0.00065).(C-D) In the Longitudinal Plasma Levels Before Diagnosis, CTSS (C) shows no significant divergence between the groups over time (P = 0.748). However, CKAP4 (D) demonstrates a significant difference in protein level trajectories between cases and controls (P = 1.84 x 10^ -8).

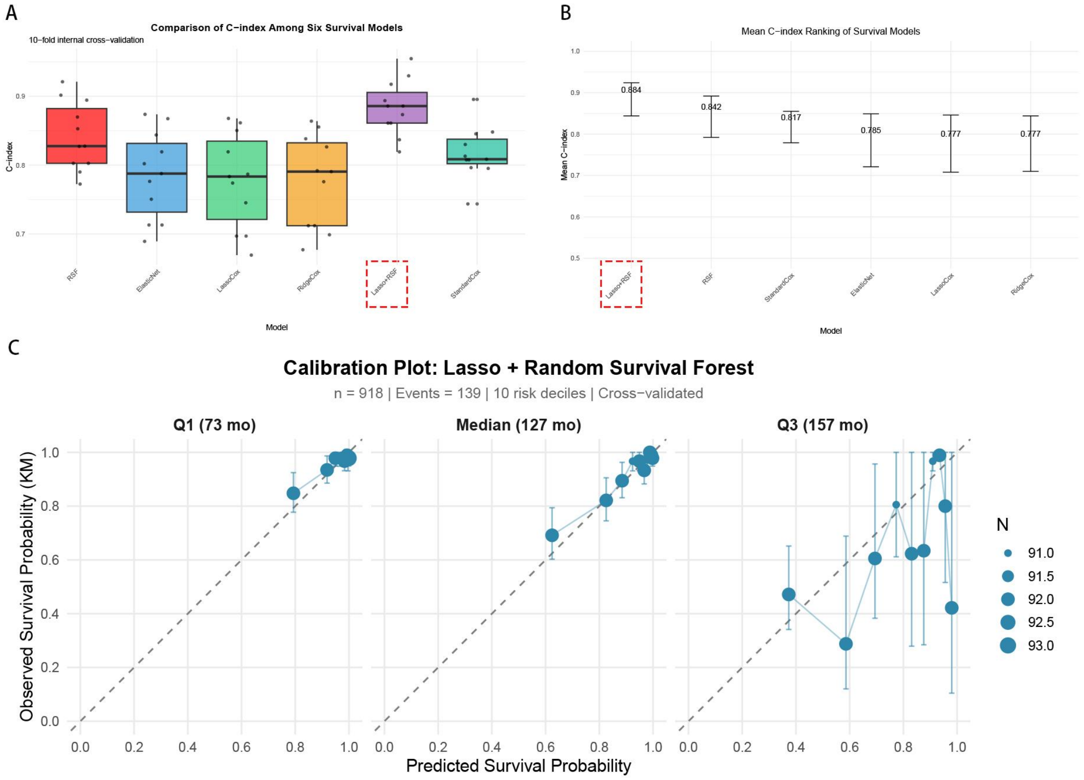

Supplementary Fig. 5 Evaluation and calibration of the predictive model.(A) Comparison of C-Index Among Six Survival Models. This panel displays the distribution of C-index values obtained through 10-fold nested internal cross-validation for six different survival algorithms: Random Survival Forest (RSF), ElasticNet, Lasso-Cox, Ridge-Cox, Lasso + RSF, and Standard Cox. The boxplots illustrate the performance variability across different data folds, with the Lasso + RSF framework (highlighted in purple) showing both the highest median C-index and the narrowest interquartile range, indicating superior stability and reduced risk of overfitting. (B) Mean C-index Ranking of Survival Models. The bar chart summarizes the average predictive performance of the evaluated models. Lasso + RSF ranks first with a mean C-index of 0.884, significantly outperforming the standalone RSF (0.842) and other penalized Cox models. Error bars represent the standard deviation across the cross-validation folds, further confirming the consistency of the integrated machine learning approach. (C) Calibration Plots for the Lasso + Random Survival Forest Model. The plots compare predicted versus observed survival probabilities at the first quartile (Q1: 73 months), median (127 months), and third quartile (Q3: 157 months). The model demonstrates robust calibration during the early-to-mid follow-up periods, while increased variance is observed at the 157-month mark, reflecting the statistical challenges associated with sparse data at the tail end of the survival curve.

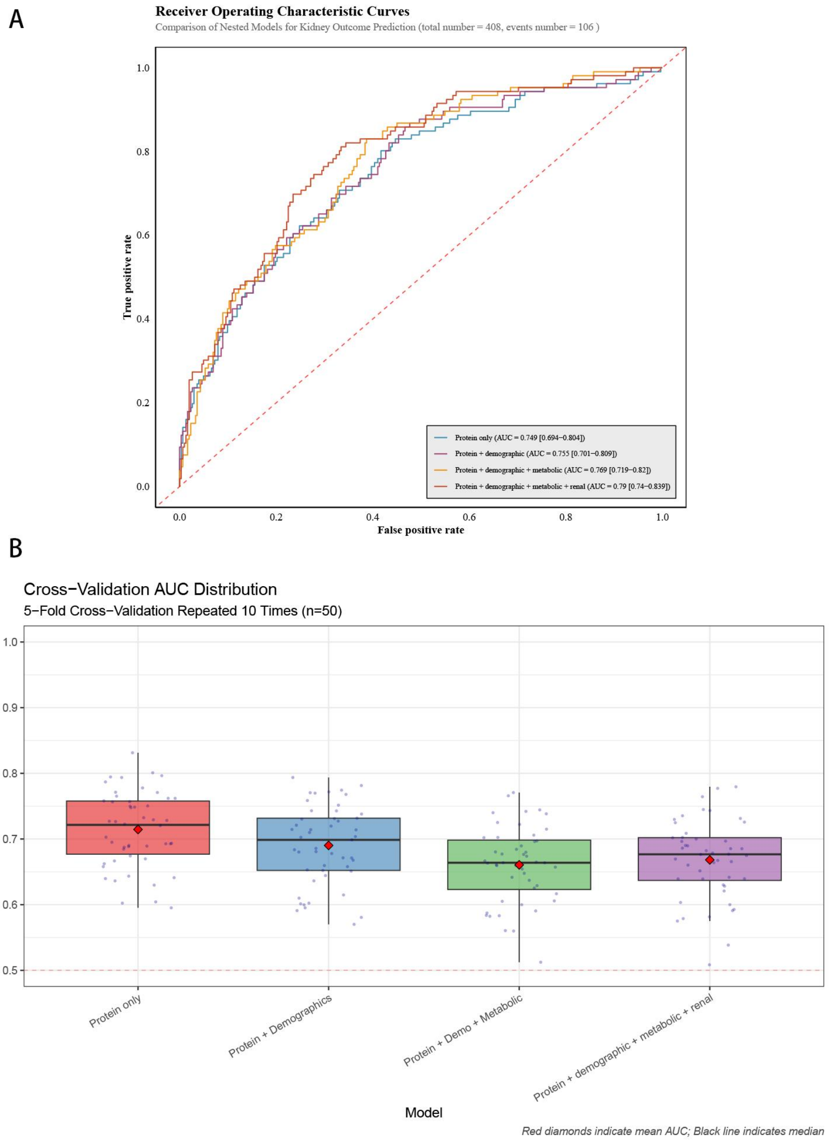

Supplementary Fig. 6 Sensitivity Analysis in the KDIGO-Defined DKD Subgroup.(A) Receiver Operating Characteristic (ROC) Curves for Kidney Outcomes. The total number was 408, and 106 people experienced the renal endpoint event. The protein signature maintained high discriminative power for kidney outcomes (AUC = 0.749[0.694, 0.804]). (B) Model Stability Assessment. Boxplots display the distribution of AUC values obtained from 50 iterations of 10-fold cross-validation for the four evaluated models. the Protein-only model demonstrated even higher and more stable AUC values, reinforcing its superior generalizability.
